# Genetic evidence for causal roles of circulating proteins on breast cancer susceptibility

**DOI:** 10.1016/j.isci.2026.115286

**Published:** 2026-03-07

**Authors:** Hanghang Chen, Qi Liu, Huiduo Zhao, Xufeng Cheng

**Affiliations:** 1 Breast Surgery, The First Affiliated Hospital of Henan University of Traditional Chinese Medicine, Zhengzhou 450000, China

**Keywords:** health sciences, medicine, medical specialty, health informatics, internal medicine, oncology

## Abstract

Circulating proteins represent promising candidates for understanding breast cancer (BC) etiology. This study employed a two-sample Mendelian randomization framework to investigate the potential causal relationships between genetically predicted levels of circulating proteins and BC risk. By integrating large-scale protein quantitative trait loci (pQTL) data from two major cohorts (DeCODE and UK Biobank) with BC genetic association data from three independent sources (FinnGen, BCAC), the analysis identified four proteins—intestinal alkaline phosphatase (ALPI), coiled-coil domain containing 134 (CCDC134), cadherin 1 (CDH1), and ST3 beta-galactoside alpha-2,3-sialyltransferase 2 (ST3GAL2)—with levels associated with a significantly reduced risk of BC. Colocalization analysis further supported a shared causal variant for ALPI. These proteins exhibited distinct associations with BC molecular subtypes. The findings highlight specific circulating proteins as potential mediators of BC risk. This work suggests avenues for exploring the biological mechanisms of BC and may inform future strategies for risk assessment.

## Highlights

• Identifies ALPI and CDH1 as causal proteins that lower breast cancer risk
• Provides genetic evidence linking four proteins (ALPI, CCDC134, CDH1, ST3GAL2) to mediation
• Integrates multi-ancestry pQTL with large-scale breast cancer GWAS for discovery
• Reveals distinct *cis*- and *trans*-pQTL mechanisms underlying the protective effects

## Graphical abstract

**Figure:**
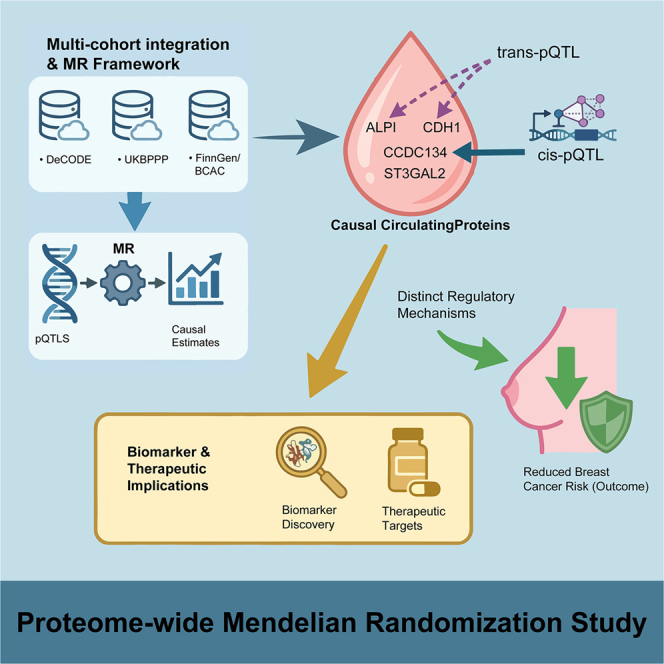

## Introduction

Breast cancer (BC) represents the most prevalent malignancy among females globally, as evidenced by 2020 cancer burden data.^1^ Accurate screening strategies are essential for identifying high-risk populations. Concurrently, population-based interventions targeting modifiable risk factors could reduce BC incidence. Circulating proteins serve as critical cancer biomarkers, with quantitative alterations reflecting physiological status and health trajectories.^2^ These biomarkers may also indicate early tumorigenesis, invasive progression, and metastatic potential.^3^

Previous studies have reported associations between circulating proteins and the pathogenesis of various cancers, including prostate,^4^ colorectal,^5^ lung,^6^ and esophagus cancer.^7^ For instance, proteins like carcinoembryonic antigen (CEA) and cancer antigen 15-3 (CA15-3) have been used for monitoring BC progression, though their utility in early detection is limited by low specificity and sensitivity for low-volume disease. Beyond these classical markers, research is increasingly focused on the cancer “secretome”—proteins secreted by tumor cells or the tumor microenvironment—which can promote processes like angiogenesis, immune evasion, and metastasis, making them promising candidate biomarkers.^8^^,^^9^ However, large-scale studies investigating causal relationships between circulating proteins and BC remain limited. Therefore, elucidating these associations could enhance diagnostic and therapeutic strategies for BC.

Mendelian randomization (MR), an epidemiological approach that utilizes genetic variants as instrumental variables, enables causal inference between exposures and disease outcomes.^10^ This method offers substantial advantages over observational studies by minimizing confounding from environmental and behavioral factors. MR strengthens causal inference through its ability to reduce residual bias, thereby improving the reliability of etiological investigations. Furthermore, this approach facilitates the identification of relationships among genetic variants, modifiable risk factors, and clinical endpoints through robust instrumental variable analysis. Zhang et al.^11^ employed MR and co-localization methods to explore actionable proteins in BC susceptibility. However, a key distinction of our study lies in its broader, agnostic screening approach; whereas Zhang et al. focused on a pre-specified set of “druggable” targets, our analysis interrogates a much wider panel of circulating proteins, allowing for identification of causal factors beyond the currently known therapeutic landscape.

Our study utilized four data sources (two exposure and two outcome datasets) within a two-sample MR (TSMR) framework to evaluate causality between genetically predicted circulating proteins and BC susceptibility/risk. This multi-dataset design, coupled with cross-validation and meta-analysis, significantly enhances the robustness and generalizability of our findings compared to analyses relying on a single source. We identified genetically predicted CDH1 (E-cadherin) and ALPI (alkaline phosphatase, intestinal) as robustly associated with decreased BC risk. These findings not only corroborate the known tumor-suppressive role of cadherin 1 (CDH1) but also highlight ALPI as a previously less explored potential player in BC pathogenesis, a protein not prominently reported in prior MR investigations. Additionally, our analyses provided genetic and MR evidence linking four circulating proteins to BC susceptibility. These results advance the genetic understanding of BC pathogenesis while offering potential targets for diagnostic and therapeutic development.

## Results

**Figure 1:**
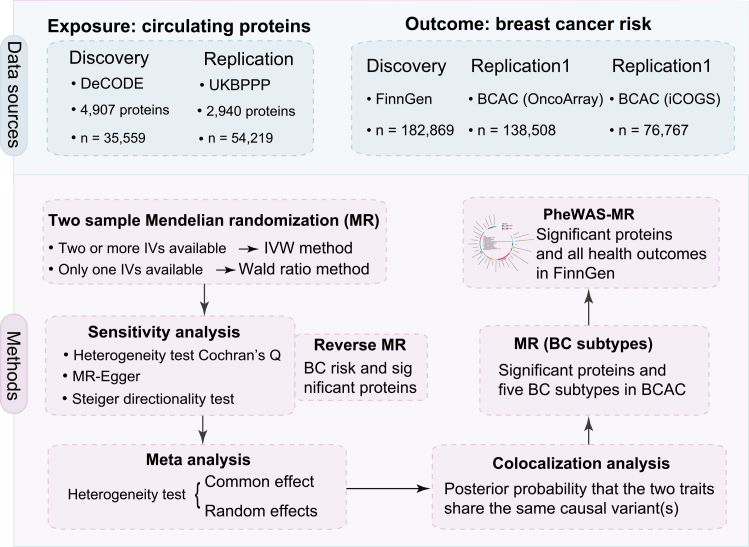
Study design overview Large-scale pQTL data from DeCODE and UKBPPP served as exposures. BC risk GWAS datasets from BCAC (OncoArray), BCAC (iCOGS), and FinnGen were analyzed as outcomes. A two-sample Mendelian randomization (MR) framework was implemented, supplemented by meta-analysis to enhance robustness. Abbreviations: pQTL, protein quantitative trait locus; GWAS, genome-wide association study; UKBPPP, UK Biobank Proteomics Project; BC, breast cancer.

The overall study design is depicted in Figure 1. A TSMR framework was employed to infer the causal associations between exposure (circulating proteins) and outcomes (BC risk). To enhance the robustness and generalizability of our findings, we implemented a rigorous discovery-replication strategy utilizing independent datasets, and meta-analyses were performed to ensure the stability of all associations.

### Six circulating proteins from the DeCODE discovery cohort are significantly associated with BC risk

**Figure 2:**
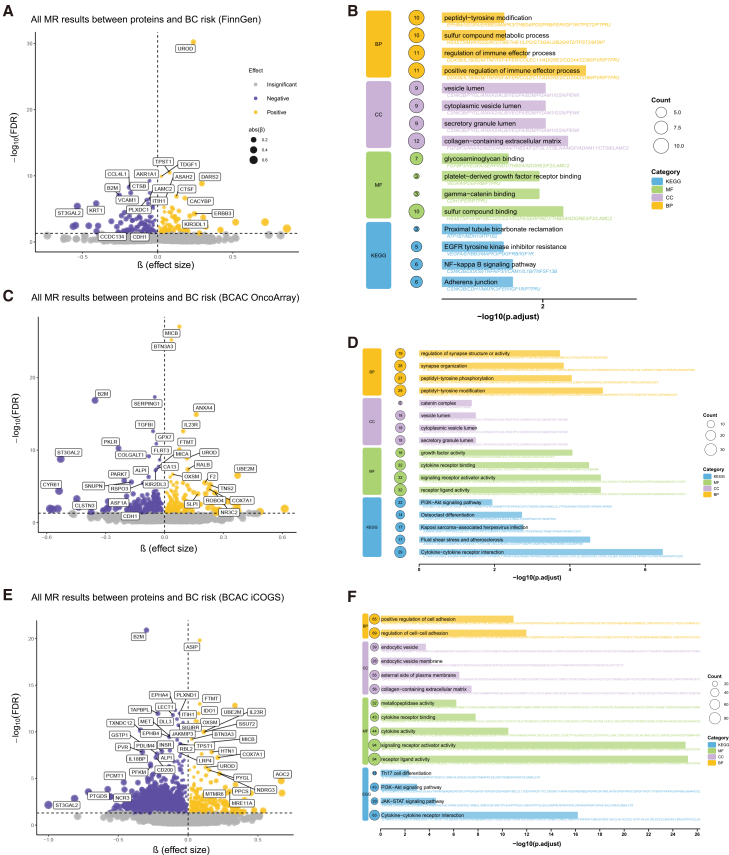
MR analysis and functional enrichment of circulating proteins associated with BC risk (A, C, and E) Volcano plots depicting the association between circulating proteins and BC risk across three independent datasets. (A) Proteins tested against BC risk using the FinnGen dataset. (C) Analysis of proteins associated with BC risk using the BCAC OncoArray dataset. (E) Proteins associated with BC risk using the BCAC iCOGS dataset. In all volcano plots, the *x* axis represents the effect size (β), and the *y* axis represents the statistical significance expressed as −log_10_ (FDR-adjusted *p* value). Each point represents a protein, with its size proportional to the absolute value of its effect size (|β|). Yellow points indicate proteins with a positive effect (risk-increasing), while purple points indicate proteins with a negative effect (risk-decreasing) on BC risk. Proteins with an FDR-adjusted *p* value <0.05 were considered statistically significant and are highlighted. (B, D, and F) Bubble charts summarizing functional enrichment analysis results for proteins significantly associated with BC risk. (B) Enrichment results for proteins associated with BC risk from the FinnGen dataset. (D) Enrichment results for proteins associated with BC risk from the BCAC OncoArray dataset. (F) Enrichment results for proteins associated with BC risk from the BCAC iCOGS dataset. For all bubble charts, the *y* axis displays the significantly enriched Gene Ontology (GO) terms or Kyoto Encyclopedia of Genes and Genomes (KEGG) pathways. The *x* axis represents the length of −log_10_ (adjusted *p* value), indicating the enrichment significance. The size of each bubble corresponds to the number of genes/proteins enriched in the respective term or pathway.

We began our analysis by screening for potential causal associations between genetically predicted levels of circulating proteins and BC risk. Through linkage equilibrium (LD) clumping, 574 proteins were excluded due to the absence of valid instrumental variables (IVs). The remaining 4,333 circulating proteins were tested against BC risk using the FinnGen dataset. Significant associations and sensitivity analysis results for these proteins are presented in Table S1. After false discovery rate (FDR) correction, 128 circulating proteins passed sensitivity tests and demonstrated causal associations with BC. The effect sizes and FDR-corrected *p* values for all proteins associated with BC risk are summarized in Figure 2A, providing a comprehensive overview of the initial screening results.

Subsequent analyses using the BCAC OncoArray dataset (Table S2) revealed that 436 circulating proteins exhibited significant causal associations with BC risk after FDR adjustment and sensitivity analyses (Figure 2C). For the BCAC iCOGS dataset (Table S3), an even larger number of proteins (1,072) showed causal associations with BC following FDR correction, with results visualized in Figure 2E. To identify the most robust and consistent protein candidates, we took the intersection of the significant hits from these three independent MR analyses. This stringent approach identified six circulating proteins that were significantly associated with BC risk across all datasets, highlighting their high potential as causal factors.

### Functional enrichment analysis of BC-associated proteins reveals key biological pathways

To gain biological insights into the functions of the proteins associated with BC risk in each dataset, we performed a functional enrichment analysis. The functional enrichment results for proteins associated with BC from the FinnGen, BCAC OncoArray, and BCAC iCOGS datasets are presented in Figures 2B, 2D, and 2F, respectively. Overall, the functional enrichment analysis of proteins significantly associated with BC risk across three independent datasets revealed both subtype-specific and consensus biological pathways, underscoring the interplay between tumor microenvironment remodeling and immune dysregulation. Functional enrichment analysis of proteins significantly associated with BC risk across three independent datasets revealed both subtype-specific and consensus biological pathways, underscoring the interplay between tumor microenvironment remodeling and immune dysregulation. Proteins linked to FinnGen-derived BC risk were enriched in immune effector processes and NF-κB signaling. Conversely, BCAC OncoArray and iCOGS proteins emphasized peptidyl-tyrosine modification, cytokine-receptor interactions, and PI3K-Akt signaling—pathways recurrently implicated in luminal and basal-like subtypes through phosphoproteomic analyses.^12^^,^^13^

Notably, the consistent enrichment of collagen-containing extracellular matrix and receptor ligand activity across all datasets reinforces the critical role of stromal remodeling and cytokine-driven crosstalk in BC progression, a pattern corroborated by integrated proteogenomic studies of tumor microenvironment dynamics.^14^

Our analysis identifies NF-κB signaling as a FinnGen-specific pathway, suggesting cohort-specific immune activation patterns, while the convergence of PI3K-Akt signaling in BCAC cohorts underscores its centrality in BC etiology. These findings not only validate established mechanisms but also reveal dataset-specific biological biases, advocating for subtype-tailored therapeutic strategies targeting immune-stromal interactions.

### Meta-analysis confirmed that four circulating proteins are significantly associated with BC risk

To obtain overall effect estimates for the proteins identified in the discovery phase, we performed a meta-analysis combining the results from the three genome-wide association study (GWAS) sources (FinnGen, BCAC OncoArray, and BCAC iCOGS). Cochran’s Q test and Higgins’s I^2^ test were used to test the heterogeneity between studies. If *p* < 0.05 or I^2^ >50%, heterogeneity was considered to exist among studies, and a random-effects model was used; otherwise, a fixed-effects model was selected as the main meta method. The existence of significant heterogeneity was acknowledged among studies investigating ALPI and BC risk (Cochran’s Q *p* value <0.05), necessitating the application of a random-effects model. In contrast, no substantial heterogeneity was detected among studies investigating coiled-coil domain containing 134 (CCDC134), CDH1, and ST3 beta-galactoside alpha-2,3-sialyltransferase 2 (ST3GAL2) (Cochran’s Q *p* value >0.05; I^2^ < 50%); thus, a fixed-effect model was applied for these proteins.

**Figure 3:**
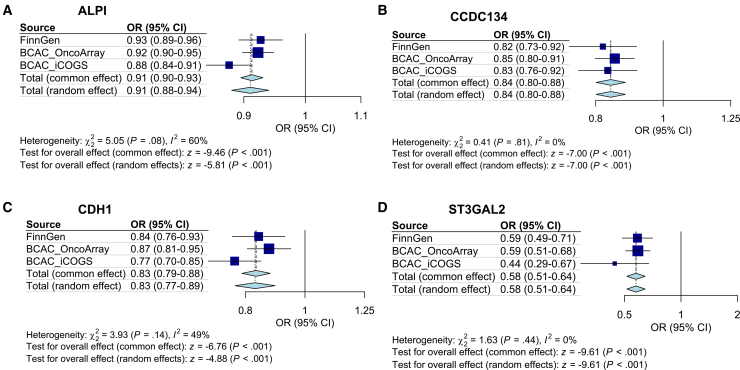
Meta-analysis of circulating proteins associated with BC risk ALPI (A), CCDC134 (B), CDH1 (C), and ST3GAL2 (D) demonstrated consistent inverse associations with BC risk. Heterogeneity was assessed using Cochran’s Q and Higgins’ I^2^ tests. Random-effects models were applied when heterogeneity was detected (*p* < 0.05 or I^2^ > 50%); otherwise, fixed-effects models were used. Data are presented as odds ratios (ORs) with 95% confidence intervals (CIs). The statistical significance of the pooled OR was assessed using a Z-test derived from the meta-analysis model.

After meta-analysis, we found that four circulating proteins are associated with reduced BC risk—ALPI (Figure 3A, odds ratio [OR] = 0.91, 95% confidence interval [CI]: 0.88–0.94, *p* < 0.001), CCDC134 (Figure 3B, OR = 0.84, 95% CI: 0.80–0.88, *p* < 0.001), CDH1 (Figure 3C, OR = 0.83, 95% CI: 0.79–0.88, *p* < 0.001), and ST3GAL2 (Figure 3D, OR = 0.58, 95% CI: 0.51–0.64, *p* < 0.001). The significant MR results between the four proteins (DeCODE) and BC risk are summarized in Table 1. Conversely, two proteins, GPX7 (Figure S1A) and TBPL1 (Figure S1B), did not show statistically significant associations with BC risk in the meta-analysis and were not pursued further. Summary information regarding the instrumental variables—such as R^2^ values and F-statistics—for the four proteins (ALPI, CCDC134, CDH1, and ST3GAL2) that showed significant associations is provided in Table S4. All instruments for our significant proteins demonstrated strong F-statistics (F > 30, far exceeding the conventional threshold of 10), effectively minimizing the risk of weak instrument bias.

**Table 1:** Significant Mendelian randomization results between proteins (DeCODE) and BC risk

Exposure	Outcome	OR	lci95	uci95	Q_pval	ple_pval	Pval	FDR
ALPI	FinnGen	0.926	0.892	0.962	0.668	0.987	6.24E−05	0.003003
	BCAC_OncoArray	0.922	0.898	0.947	0.548	0.983	2.42E−09	5.00E−07
	BCAC_iCOGS	0.878	0.844	0.913	0.133	0.200	5.95E−11	9.94E−09
CCDC134	FinnGen	0.819	0.733	0.916	0.175	0.716	0.000466	0.015301
	BCAC_OncoArray	0.852	0.799	0.909	0.539	0.563	1.28E−06	0.000109
	BCAC_iCOGS	0.833	0.756	0.917	0.264	0.386	0.000211	0.002006
CDH1	FinnGen	0.841	0.762	0.928	0.752	0.574	0.000554	0.017264
	BCAC_OncoArray	0.874	0.806	0.948	0.059	0.884	0.001229	0.015458
	BCAC_iCOGS	0.768	0.696	0.848	0.224	0.103	1.73E−07	1.10E−05
ST3GAL2	FinnGen	0.586	0.486	0.706	0.999	0.968	1.73E−08	3.94E−06
	BCAC_OncoArray	0.589	0.507	0.685	0.724	0.404	4.97E−12	1.66E−09
	BCAC_iCOGS	0.444	0.292	0.673	NA	NA	0.000135	0.001439

OR, odds ratio; lci95, lower 95% confidence interval; uci95, upper 95% confidence interval; Q_pval, *p* value of Cochran’s Q statistics; ple_pval, *p* value of pleiotropy test; FDR, false discovery rate; ALPI, alkaline phosphatase, intestinal; CCDC134, coiled-coil domain containing 134; CDH1, cadherin 1; ST3GAL2, ST3 beta-galactoside alpha-2,3-sialyltransferase 2.

### Distinct causal mechanisms of BC risk predicted by *cis*- and *trans*-pQTLs for four circulating proteins

**Figure 4:**
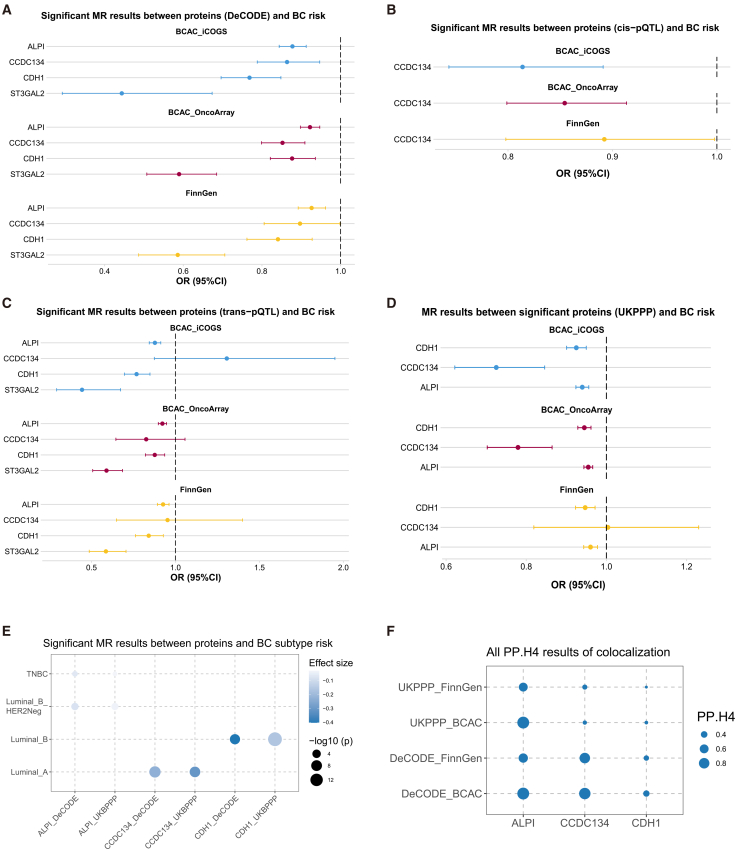
MR associations between proteins and BC risk (A) Forest plot displays odds ratios (ORs) with 95% confidence intervals (CIs) for four circulating proteins (DeCODE dataset) across three BC outcomes. (B) Forest plot of the association between BC risk and CCDC134, instrumented exclusively by *cis*-pQTLs. (C) Forest plot of the causal effects of ALPI, CDH1, and ST3GAL2 on BC risk, instrumented solely by *trans*-pQTLs. (D) Forest plot illustrates ORs and 95% CIs for three proteins (UKBPPP dataset) versus BC outcomes. (E) Protein-BC subtype associations: color intensity denotes effect direction (red: risk elevation; blue: protection), while dot size reflects association significance (−log10[*p*]). (F) Colocalization analysis identifies shared genetic variants between ALPI and BC risk, with PP.H4 indicating posterior probability for shared causal variants.

ALPI, CCDC134, CDH1, and ST3GAL2 are associated with reduced BC risk (Figure 4A). To delineate the distinct genetic mechanisms underpinning the causal associations, we conducted stratified MR analyses using *cis*- and *trans*-pQTLs as instrumental variables for the four significant proteins (ALPI, CCDC134, CDH1, and ST3GAL2). When instrumented exclusively by *cis*-pQTLs, the protective effect of CCDC134 against BC risk remained significant, confirming that its association is mediated through local genetic regulation proximal to its encoding gene (Figure 4B). Conversely, analyses utilizing only *trans*-pQTLs demonstrated that ALPI, CDH1, and ST3GAL2 consistently significantly reduced BC risk (Figure 4C). This indicates that the causal effects of these three proteins are driven by genetic variants acting in *trans*, likely reflecting broader, potentially master-regulatory, biological pathways influencing both protein abundance and disease pathogenesis. The stratification of pQTL types thus reveals that the robust causal associations identified for the four proteins are mediated by two distinct mechanistic paths: a *cis*-acting mechanism for CCDC134 and *trans*-acting mechanisms for ALPI, CDH1, and ST3GAL2, reinforcing the validity of the findings and providing deeper etiological insights.

### Replication in the UKBPPP cohort validates associations for ALPI and CDH1

To independently validate the causal associations identified in the DeCODE cohort, we sought to replicate our findings using pQTL data from the UK Biobank Plasma Proteomics Project (UKBPPP). Among these, three significant proteins—ALPI, CCDC134, and CDH1—could be found in the UKBPPP dataset and were tested for validation. Notably, ALPI and CDH1 from the UKBPPP dataset were also significantly associated with BC risk in the replication analysis (Figure 4D; Table 2), providing strong support for their robust causal roles. The association for CCDC134, however, did not reach statistical significance in the UKBPPP replication cohort, suggesting that this association may require further investigation in even larger samples.

**Table 2:** MR results between significant proteins (UKPPP) and BC risk

Exposure	Outcome	OR	lci95	uci95	Pval	FDR
ALPI	FinnGen	0.960	0.943	0.978	8.86E−06	1.33E−05
	BCAC_OncoArray	0.955	0.944	0.966	3.48E−15	3.13E−14
	BCAC_iCOGS	0.940	0.924	0.956	9.91E−13	4.46E−12
CCDC134	FinnGen	1.004	0.819	1.230	0.971939	0.971939
	BCAC_OncoArray	0.779	0.703	0.864	2.42E−06	4.35E−06
	BCAC_iCOGS	0.725	0.622	0.846	4.32E−05	4.86E−05
CDH1	FinnGen	0.947	0.923	0.972	3.37E−05	4.33E−05
	BCAC_OncoArray	0.945	0.929	0.961	1.77E−10	5.32E−10
	BCAC_iCOGS	0.925	0.901	0.950	8.09E−09	1.82E−08

### Reverse MR analysis indicates no reverse causality from BC risk to protein levels

To examine the possibility of reverse causality—where BC status influences circulating protein levels rather than the other way round—we performed reverse MR analyses. In these analyses, BC was set as the exposure, and the identified causal proteins were set as outcomes. Overall, we found no robust evidence for reverse causal associations between BC risk and the three significant proteins—ALPI, CCDC134, and CDH1. There were a few exceptions, such as a nominally significant causal association between BC risk (FinnGen and BCAC) and CCDC134 (UKPPP) and a causal association between BC risk (BCAC) and CDH1 (UKPPP). However, these isolated findings were inconsistent across datasets and did not survive multiple testing correction; thus, they are not considered robust. The reverse MR results between BC risk and three significant proteins were showed in Figure S2.

### Differential associations between three candidate proteins and BC molecular subtypes

Given the heterogeneity of BC, we explored the potential causal associations between the three validated proteins (ALPI, CCDC134, and CDH1) and the risks of specific BC molecular subtypes (luminal A-like, luminal B/HER2-negative-like, luminal B-like, HER2-enriched-like, and triple-negative/basal-like). Potential causal associations were identified between three proteins and BC subtype risk (Figure 4E). Specifically, ALPI is negatively associated with luminal B (HER2−) and triple-negative BC (TNBC) subtype risk, suggesting a broader protective role across major subtypes. CCDC134 is only associated with reduced luminal A subtype risk, indicating a potentially subtype-specific function. CDH1 could potentially reduce the luminal B subtype risk. These differential association patterns hint at the distinct biological roles these proteins may play in different BC contexts.

### Colocalization analysis indicates shared genetic variants between ALPI and BC risk

To verify that the observed MR association between ALPI and BC risk was not driven by LD between distinct causal variants for each trait, we performed genetic colocalization analysis. Colocalization analysis can verify whether there are shared variants between traits in the specified gene region. A posterior probability for hypothesis 4 (PPH4) >0.8 means strong colocalization evidence. Among the 3 proteins that had causal associations with BC risk, ALPI was found to have strong colocalization evidence with BC risk (PPH4 > 0.8, Figure 4F). This finding increases our confidence that the genetic association signal for ALPI and BC risk originates from the same causal variant(s), strengthening the inference of a direct causal relationship.

### PheWAS-MR analysis reveals the pleiotropic effects of ALPI

To comprehensively map the potential functions and side effects of ALPI, which demonstrated a robust causal relationship with BC risk, we conducted a phenome-wide MR (PheWAS-MR) analysis. PheWAS analysis of ALPI, which demonstrated a robust causal relationship with BC risk, revealed significant associations with 31 diverse health outcomes after multiple testing correction. The strongest associations were observed with K11_PANCOTH (pancreatic disorders), RX_INFERTILITY (treatment for infertility), and AB1_SEXUAL_TRANSMISSION (sexual transmission-related conditions). Notably, ALPI was linked to an elevated risk of vascular disorders of the intestines, bronchiectasis, and malignant neoplasms of the esophagus.

**Figure 5:**
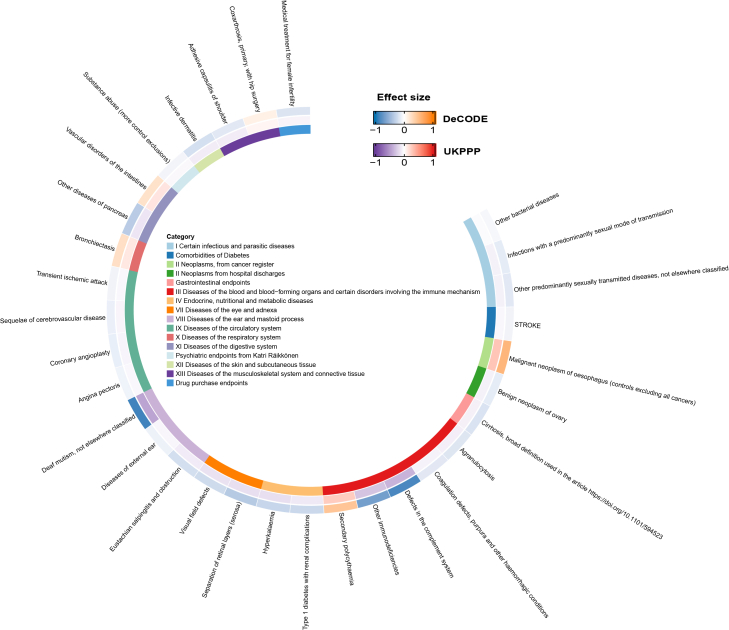
Phenome-wide associations of ALPI Ring heatmap demonstrates ALPI’s genetic links to 31 phenotypes across 27 disease categories. Warm hues (orange/red) indicate positive associations for DeCODE and UKBPPP datasets, respectively; cool tones (blue/purple) represent inverse relationships. Effect sizes are proportional to color intensity.

These findings align with existing literature highlighting ALPI’s role in intestinal barrier integrity, Lipopolysaccharide (LPS) detoxification, and inflammation resolution.^15^^,^^16^ While prior studies have established ALPI’s involvement in mucosal protection and metabolic homeostasis, our analysis provides genetic evidence of its broad pleiotropic effects, particularly implicating ALPI in pathways shared across gastrointestinal, respiratory, and neoplastic diseases. This underscores ALPI’s systemic influence beyond BC, suggesting its potential as a therapeutic target or biomarker for multiple conditions. The significant associations and the sensitivity analysis results were shown in Figure 5 and Table S5.

## Discussion

Circulating proteins have emerged as promising candidates for pre-diagnostic malignancy detection, offering a non-invasive window into early cancer pathogenesis.^17^ Our proteome-wide MR analysis, which investigated the causal effects of over 4,000 circulating proteins on BC risk, aligns with growing evidence that serum protein profiles can be altered years before clinical diagnosis. By integrating large-scale pQTL data from sources like DeCODE and UKBPPP with BC GWAS from multiple cohorts, we identified genetically proxied CDH1 and ALPI as robust protective factors against BC risk. This approach leverages the causal inference strength of MR to minimize confounding and reverse causality, providing reliable insights into potential biomarkers. Notably, our findings resonate with studies highlighting proteins as direct indicators of cellular activity and disease mechanisms, underscoring their superiority over DNA or RNA in reflecting real-time pathological processes. The consistency of these results across independent datasets and their validation through sensitivity analyses strengthen the hypothesis that circulating proteins play an etiological role in BC development, potentially enabling early-risk stratification and intervention.

The protective effect of genetically proxied CDH1 against BC risk corroborates its well-established role as a tumor suppressor encoding E-cadherin, a key mediator of cell-cell adhesion. Loss of CDH1 function impairs epithelial integrity, facilitating cancer cell detachment and metastasis—a mechanism particularly relevant in invasive lobular BC.^18^ While CDH1 mutations are implicated in familial gastric,^19^ diffuse gastric, colorectal signet-ring cell, and lobular BC,^20^ our findings align with evidence linking CDH1 downregulation to BC invasion and metastasis.^21^ Notably, our MR estimates for CDH1 were consistent across multiple BC GWAS sources, and the association was replicated in the UKBPPP dataset, enhancing the robustness of this finding beyond single-cohort analyses. Future studies should explore whether CDH1’s effects are modulated by hormonal pathways or BC subtypes, given the heterogeneity of BC molecular profiles.

ALPI represents a previously less explored protective factor in BC etiology, as its role in mammary carcinogenesis had been largely unexplored prior to our study. Interestingly, ALPI was not identified as a significant hit in the MR analysis by Zhang et al.,^11^ highlighting the value of our broader proteomic screening approach in uncovering associations beyond traditionally studied druggable targets. ALPI is primarily known for its role in intestinal barrier maintenance, lipopolysaccharide detoxification, and anti-inflammatory responses, with animal models demonstrating its critical function in preserving gut integrity and mitigating systemic inflammation.^22^^,^^23^ Our MR analysis suggests that these immunomodulatory properties may extend to BC prevention, possibly via dampening chronic inflammation or regulating microbiome-derived oncogenic signals. For instance, elevated ALPI levels could inhibit Toll-like receptor signaling or reduce oxidative stress, pathways implicated in BC progression. This finding gains support from studies linking ALPI deficiency to metabolic and cardiovascular diseases, indicating its broad systemic impact.^24^ However, the PheWAS results showing ALPI’s association with increased risks of vascular intestinal disorders and esophageal malignancies highlight potential trade-offs, echoing concerns that enhancing protein levels for therapeutic benefits might inadvertently promote off-target effects. Further research is needed to dissect ALPI’s mechanism in BC, particularly its interaction with estrogen receptor pathways or immune cell infiltration in the tumor microenvironment.

Our study underscores the utility of integrating MR with colocalization and sensitivity analyses to address methodological challenges in causal inference. The relaxed LD clumping criteria (r^2^ < 0.1, 100 kb) for protein instruments accounted for the less complex LD patterns of pQTLs compared to disease-related variants, thereby maximizing statistical power while controlling for confounding. This approach is consistent with methodological recommendations for protein MR studies and helps retain stronger instruments for proteins with simpler LD patterns. Moreover, the replication of ALPI and CDH1 associations in independent cohorts (e.g., UKBPPP) enhances reliability. The convergence of our findings with functional enrichment analyses—which implicated immune and metabolic pathways—further aligns with systems biology perspectives that prioritize proteins as hubs in disease networks. Nonetheless, we acknowledge that MR estimates may still be influenced by horizontal pleiotropy or population-specific biases, necessitating complementary approaches like colocalization to validate shared causal variants (e.g., the strong evidence for ALPI and BC risk in designated genomic regions).

Our findings should be interpreted within the context of a rapidly evolving landscape of proteome-wide MR studies, which, while employing similar frameworks, often yield distinct protein candidates due to methodological heterogeneity. For instance, Mälarstig et al.,^25^ utilizing the Olink PEA Explore platform in a female-specific cohort (*n* = 598), identified five plasma proteins (CD160, DNPH1, LAYN, LRRC37A2, and TLR1) with potential causal roles in BC risk. Notably, TLR1 was also implicated in other independent investigations, reinforcing its potential involvement in BC pathogenesis through innate immune pathways.^26^ Similarly, a more recent study by Yao et al.,^27^ which integrated pQTLs from 2,004 circulating proteins, highlighted TLR1, A4GALT, SNUPN, and CTSF as potential drug targets, suggesting a convergence on specific immune-related and cellular maintenance pathways despite differing protein panels. The discrepancy in specific protein hits between our study (which identified ALPI and CDH1) and others primarily stems from key methodological variations, including the use of different proteomic platforms (SomaScan vs. Olink), which capture distinct but overlapping sets of proteins with varying affinities; substantial differences in sample sizes of the pQTL discovery cohorts; and variations in the definitions of BC outcomes and subtypes across the source GWAS.

Importantly, the robust genetic evidence provided by this comprehensive TSMR framework, with the consistent validation of ALPI and CDH1 as protective factors across multiple independent datasets and proteomic platforms, paves the way for clinical applications. The identification of ALPI extends its known role in gut barrier function to BC etiology, suggesting systemic immunomodulatory mechanisms, while the reaffirmation of CDH1’s tumor-suppressive function highlights the precision of our approach. These proteins could be integrated into existing biomarker panels (e.g., CA15-3 or CEA) to enhance BC screening specificity, particularly for aggressive subtypes like TNBC. Prospective trials should validate their utility in liquid biopsy-based early detection, leveraging technologies such as SomaScan or Olink assays. Ultimately, bridging these genetic discoveries to therapeutic strategies—for instance, developing ALPI-enhancing interventions or CDH1-stabilizing compounds—could transform BC prevention. Future research should prioritize *trans*-ethnic replication, incorporate functional assays to elucidate mechanistic pathways, and explore the utility of these proteins in risk stratification models or as therapeutic targets, further emphasizing the value of proteomic MR in guiding precision oncology.

### Limitations of the study

This study has several limitations that should be considered. First, while multiple sensitivity analyses were employed to address pleiotropy, the MR approach remains susceptible to residual confounding from horizontal pleiotropy, an inherent methodological constraint. Second, the pQTL data were primarily derived from European-ancestry populations, which may limit the generalizability of our findings to other ethnic groups due to potential differences in genetic architecture.

Third, the analysis was necessarily restricted to the subset of circulating proteins assayed by the available proteomic platforms. Many low-abundance or unmeasured proteins with potential causal roles were not investigated, possibly omitting other significant candidates. Finally, the genetic instruments proxy protein abundance levels, but they may not directly reflect functional activity (e.g., ALPI’s enzymatic function). Therefore, while our results support a causal link between genetically predicted protein levels and BC risk, the precise biological mechanisms require further experimental validation.

Future studies should aim to incorporate multi-ancestry pQTL data, utilize sex-stratified analyses where possible, and employ unified proteomic platforms to enhance consistency and generalizability.

## Resource availability

### Lead contact

Further information and requests for resources and reagents should be directed to and will be fulfilled by the lead contact, Hanghang Chen (461652608@qq.com).

### Materials availability

This study did not generate new unique reagents.

### Data and code availability

• This study analyzes existing, publicly available data. The pQTL data for the discovery cohort were obtained from the DeCODE genetics study (35,559 individuals; 4,907 aptamers via SomaScan).^28^ The replication pQTL data were obtained from the UK Biobank Proteomics Project (UKBPPP, 54,219 individuals; Olink platform).^29^ BC risk GWAS summary statistics were sourced from FinnGen,^30^ BCAC OncoArray,^31^ and BCAC iCOGS.^31^
• This paper does not report original code. All analyses were performed using standard software and R packages as detailed in the STAR Methods. The specific analysis scripts are available from the lead contact upon reasonable request.
• Any additional information required to reanalyze the data reported in this paper is available from the lead contact upon request.

## Acknowledgments

We gratefully acknowledge the following sources of support that made this research possible. This study was supported by the 10.13039/501100006407Natural Science Foundation of Henan Province of China (grant no. 232300421183), the Henan Province Traditional Chinese Medicine Key Discipline Construction Project (grant no. CZ0366-07), and the Doctoral Research Foundation of the First Affiliated Hospital of 10.13039/100017634Henan University of Chinese Medicine (grant no. 2024BSJJ044).

We are deeply indebted to the consortia and biobanks that provided the genetic data essential for this study: the Breast Cancer Association Consortium (BCAC), DeCODE, and UKPPP studies. We extend our sincere thanks to the investigators and participants of all these studies, without whom this large-scale genetic research would not be feasible. We specifically acknowledge the contributions of the FinnGen consortium for making their R9 release data publicly available.

## Author contributions

H.C., conceptualization, methodology, formal analysis, software, visualization, writing – original draft, and funding acquisition; Q.L., validation, data curation, formal analysis, and writing – original draft; H.Z., data curation and validation; X.C., conceptualization, methodology, writing – review and editing, and funding acquisition.

## Declaration of interests

The authors declare no competing interests.

## STAR★Methods

### Key resources table

**Table:** 

REAGENT or RESOURCE	SOURCE	IDENTIFIER
**Deposited data**		

DeCODE genetics plasma protein QTL summary statistics	Ferkingstad et al.^28^	https://doi.org/10.1038/s41588-021-00978-w
UK Biobank Plasma Proteomics Project (UKBPPP) pQTL summary statistics	Sun et al.^29^	https://doi.org/10.1038/s41586-023-06592-6
FinnGen consortium R9 release GWAS summary statistics	Kurki et al.^30^	https://doi.org/10.1038/s41586-022-05473-8
Breast Cancer Association Consortium (BCAC) OncoArray GWAS summary statistics	Zhang et al.^31^	https://doi.org/10.1038/s41588-020-0609-2
Breast Cancer Association Consortium (BCAC) iCOGS GWAS summary statistics	Zhang et al.^31^	https://doi.org/10.1038/s41588-020-0609-2

**Software and algorithms**		

R Project for Statistical Computing (v4.2.3)	R Core Team, 2022	https://www.r-project.org/
PLINK (v1.9)	Chang et al., 2015	https://www.cog-genomics.org/plink/
TwoSampleMR R package (v0.5.7)	Hemani et al., 2018	https://mrcieu.github.io/TwoSampleMR/
MR-PRESSO R package (V1.0)	Verbanck et al., 2018	https://github.com/rondolab/MR-PRESSO
coloc R package (V5.1.0)	Giambartolomei et al., 2014	https://cran.r-project.org/package=coloc
clusterProfiler R package (V4.6.2)	Wu et al., 2021	https://bioconductor.org/packages/clusterProfiler
meta R package (v6.2-1)	Balduzzi et al., 2019	https://cran.r-project.org/package=meta

### Experimental model and study participant details

This study utilized publicly available summary-level genetic data from human populations. No new primary data from human participants, animals, or cell lines were collected for this work. Therefore, details on maintenance, care, authentication, or mycoplasma testing are not applicable.

#### Human participants

All genetic association data were obtained from large-scale consortia. Detailed cohort descriptions are available in the respective original publications, the key references and data identifiers for which are listed in the key resources table.

• DeCODE pQTL cohort: The study included 35,559 individuals of European ancestry from Iceland.
• UK Biobank Proteomics Project (UKBPPP) cohort: The study included 54,219 individuals of European ancestry from the UK Biobank.
• Breast Cancer Association Consortium (BCAC) cohorts: The OncoArray and iCOGS GWAS included breast cancer cases and controls predominantly of European ancestry (total cases: ∼118,474; total controls: ∼96,201). All participants were female.
• FinnGen cohort: The FinnGen R9 release includes genetic and health data from Finnish biobank participants. The breast cancer endpoint (N14) comprised 15,680 cases and 167,189 controls. All participants are of Finnish ancestry.

#### Ethics oversight

All original studies (DeCODE, UKBPPP, BCAC, FinnGen) from which summary data were obtained received approval from their respective institutional review boards or ethics committees, and all participants provided informed consent. This secondary analysis of publicly available summary-level data required no additional ethical clearance.

#### Consideration of sex and gender

The outcome data (BCAC, FinnGen) are derived from studies of female participants, as breast cancer is the phenotype of interest. The exposure (pQTL) data from DeCODE and UKBPPP include both male and female participants, as protein levels were measured in a general population sample. Our Mendelian randomization analysis uses genetic variants as proxies for protein levels, which are not directly stratified by sex in the source pQTL studies. Therefore, while our findings are interpreted in the context of female breast cancer risk, we are unable to assess the specific influence of sex or gender on the causal estimates from the exposure side, which is a limitation of using non-sex-stratified pQTL data.

#### Human subjects allocation

As this study uses summary statistics, we did not allocate individual subjects to experimental groups. The case-control definitions and sample sizes for the breast cancer GWAS are as established and described by the original consortia (see above and key resources table).

### Method details

We employed a two-sample Mendelian Randomization (TSMR) framework^10^ to evaluate associations between genetically predicted circulating proteins and BC risk, adhering to STROBE-MR guidelines.^32^ A discovery-replication strategy was implemented.

#### Instrumental variable (IV) selection

For each protein, genetic IVs were selected as SNPs significantly associated with the exposure (*p* < 5 × 10^−8^) from the DeCODE (discovery) and UKBPPP (replication) datasets. Furthermore, to minimize the inclusion of invalid IVs that might exhibit horizontal pleiotropy through direct effects on the outcome, we required that these IVs show no strong association with the outcome in the outcome GWAS summary statistics at a threshold of *p* > 1 × 10-5. This threshold is a conventional practice in MR studies to balance the reduction of pleiotropic bias with the retention of statistical power. A relaxed LD clumping threshold (r^2^ < 0.1, 100 kb) was applied using PLINK (v1.9)^33^ to account for simpler LD patterns of pQTLs. The F-statistic for each IV was calculated to assess strength: *F* = (*N*-2) × R^2^ ÷ (1-R^2^)(R^2^: interpretability of instrumental variables, *N*: sample size) and R^2^ could be calculated using this formula: R^2^ = β^2^(1-EAF) × 2EAF(EAF: effect elle frequency, β: beta size).^34^ Conventionally, IV with *F* < 10 was defined as weak IV and was removed to avoid weak instrumental variable bias. *Cis*-pQTLs were defined as variants within ±1 Mb of the gene’s transcription start site; *trans*-pQTLs were outside this region.

#### Outcome data extraction

The selected IVs were extracted from three independent BC GWAS summary statistics (FinnGen, BCAC OncoArray, BCAC iCOGS). Proxies for missing SNPs were not sought.

#### Data harmonization

Effect alleles for exposure and outcome datasets were harmonized to ensure alignment. Palindromic SNPs were handled with caution. The Wald ratio method was used for single-IV analyses.

#### Functional enrichment analysis

Proteins significantly associated with BC risk were subjected to functional enrichment analysis using the R package clusterProfiler (v4.6.2)^35^ for Gene Ontology (GO) terms and KEGG pathways, with an FDR-adjusted *p*-value <0.05 considered significant.

#### Reverse MR analysis

To examine reverse causality, BC was set as the exposure and causal proteins as outcomes. Stringent LD clumping (r^2^ < 0.001, 10,000 kb window) was applied to BC genetic instruments (*p* < 5 × 10^−8^).

#### MR analysis of BC subtypes

Causal associations were explored between significant proteins and specific BC molecular subtypes (Luminal A-like, Luminal B/HER2-negative-like, etc.) using the same MR framework.

#### PheWAS-MR analysis

Phenome-wide MR (PheWAS-MR) was conducted between significant proteins and 2,099 health phenotypes in FinnGen R9 to assess potential on-target side effects.

### Quantification and statistical analysis

#### Primary MR analysis

The Inverse-Variance Weighted (IVW) method was the primary analytical approach.^36^ For analyses with a single IV, the Wald ratio method was applied.^37^ A false discovery rate (FDR) correction was applied using the Benjamini-Hochberg procedure; an FDR-adjusted *p*-value (q-value) < 0.05 was considered statistically significant for primary analyses.

#### Sensitivity analyses

##### Heterogeneity

Assessed using Cochran’s Q statistic (*p* < 0.05 indicating significant heterogeneity).^38^

##### Horizontal pleiotropy

Evaluated using the MR-Egger intercept test (a non-zero intercept with *p* < 0.05 suggesting potential pleiotropy)^39^ and MR-PRESSO to identify and correct for outliers.^39^

##### Directionality

The MR Steiger directionality test was used to exclude reverse causality.^40^

##### Leave-one-out analysis

The influence of individual SNPs was assessed by sequentially excluding each SNP and re-running the MR analysis.

#### Meta-analysis

Significant MR results across cohorts were meta-analyzed using the meta R package.^41^ Heterogeneity was assessed using Cochran’s Q and I^2^ statistics^42^; a random-effects model was used if *p* < 0.05 or I^2^ > 50%, otherwise a fixed-effects model was applied.

#### Colocalization analysis

The coloc R package^43^ was used to perform genetic colocalization within a ±1 Mb region around the lead pQTL. A posterior probability for hypothesis 4 (PPH4) ≥ 0.80 was considered strong evidence for a shared causal variant.^44^

#### Software

All statistical analyses were performed using R software. Key packages and versions are listed in the key resources table.

## Supplemental information

Document S1. Figures S1 and S2 and Table S4

Table S1. Full results of the Mendelian randomization analysis for circulating proteins against breast cancer risk using the FinnGen dataset, related to Figure 2A

Table S2. Full results of the Mendelian randomization analysis for circulating proteins against breast cancer risk using the BCAC OncoArray dataset, related to Figure 2C

Table S3. Full results of the Mendelian randomization analysis for circulating proteins against breast cancer risk using the BCAC iCOGS dataset, related to Figure 2E

Table S5. Full results of the phenome-wide Mendelian randomization (PheWAS-MR) analysis for ALPI, related to Figure 5
