# Supplementary material for: Genetic evidence for causal roles of circulating proteins on breast cancer susceptibility
Source: iScience. 2026 Mar 7;29(4):115286. doi: 10.1016/j.isci.2026.115286 (PMC13049426; doi:10.1016/j.isci.2026.115286)
Supplement: Document S1. Figures S1 and S2 and Table S4 [file mmc1.pdf]

iScience, Volume *29*

Supplemental information

Genetic evidence for causal roles of circulating proteins on breast cancer susceptibility

Hanghang Chen, Qi Liu, Huiduo Zhao, and Xufeng Cheng

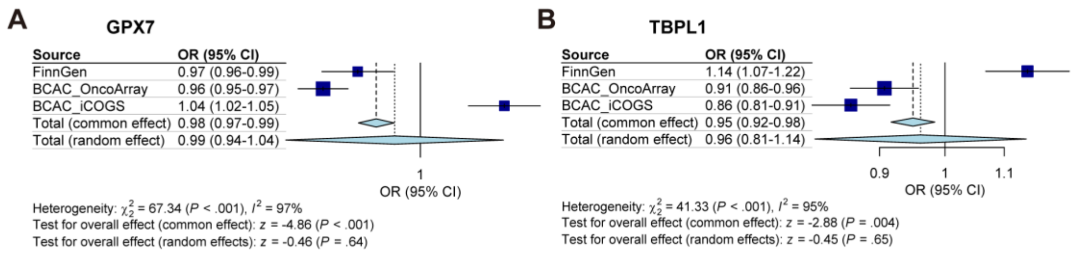

Figure S1: Meta-analysis of non-significant protein associations with breast cancer risk. (A) Forest plot for GPX7. (B) Forest plot for TBPL1. Heterogeneity was assessed using Cochran's Q and Higgins' I² tests. Random-effects models were applied when heterogeneity thresholds were met ($P < 0.05$ or $I^2 > 50\%$); otherwise, fixed-effects models were implemented.

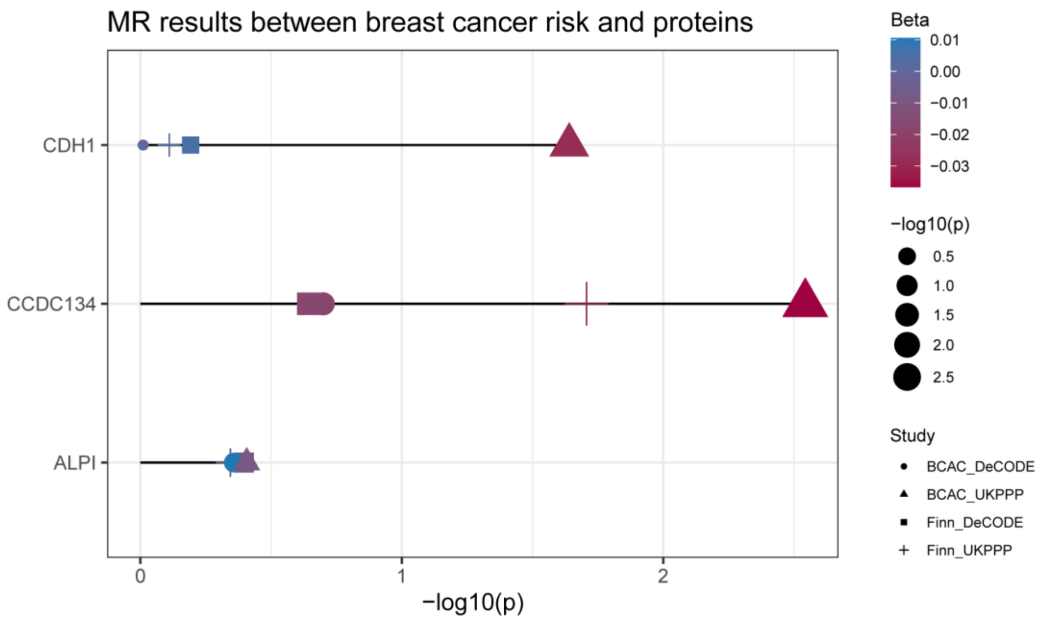

Figure S2: Reverse Mendelian randomization analysis of breast cancer risk and candidate proteins.

Dot plot visualizes effect directions (blue: $\beta > 0$; red: $\beta < 0$) and significance ($-\log_{10}$[p], dot size) for BC risk associations with three proteins across datasets. Dot shapes distinguish data sources.

Table S1: Full results of the Mendelian randomization analysis for circulating proteins against breast cancer risk using the FinnGen dataset, related to Figure 2A.

(This table is provided as a separate Excel file due to its length. See the file Table S1.xlsx.)

Table S2: Full results of the MR analysis for circulating proteins against BC risk using the BCAC OncoArray dataset, related to Figure 2C.

(This table is provided as a separate Excel file due to its length. See the file Table S2.xlsx.)

Table S3: Full results of the MR analysis for circulating proteins against BC risk using the BCAC iCOGS dataset, related to Figure 2E.

(This table is provided as a separate Excel file due to its length. See the file Table S3.xlsx.)

Table S4: Summary of instrumental variables for the four significant proteins.

Protein	SNP	effect_allele.exposure	other_allele.exposure
ALPI	rs601338	A	G
ALPI	rs576123	T	C
ALPI	rs411482	T	C
ALPI	rs17474001	T	C
ALPI	rs183853102	A	T
ALPI	rs11794634	C	T
ALPI	rs17851884	A	G
ALPI	rs72775473	A	G
ALPI	rs560066139	C	G
ALPI	rs35650232	G	T
ALPI	rs558240	A	G
ALPI	rs7033492	C	T
ALPI	rs1688263	G	A
ALPI	rs4015	T	C
ALPI	rs145526382	T	C
ALPI	rs28627930	TACCA	TACTA
ALPI	rs111277780	T	C
ALPI	rs10922098	T	C
ALPI	rs4801774	T	C
ALPI	rs113361172	A	G
ALPI	rs418821	C	G
ALPI	rs2387343	AATAATCATCATCATCATCGTCATCATCATCG	AATCATCATCATCATCAT[illegible]
ALPI	rs79198796	T	C
ALPI	rs28463601	A	G
ALPI	rs148539701	G	T
ALPI	rs28362819	T	C
ALPI	rs11523307	G	A

ALPI	rs28660186	C	A
ALPI	rs34354498	T	C
ALPI	rs144762712	TCTTTTTCTT	TCTTTTTTTT
ALPI	rs141535538	T	C
ALPI	rs11244051	A	G
ALPI	rs535015446	A	G
ALPI	rs579232	G	T
ALPI	rs3815692	A	C
ALPI	rs73053569	A	G
ALPI	rs55710199	T	C
ALPI	rs6597608	A	G
ALPI	rs112142514	G	T
ALPI	rs575111039	T	C
ALPI	rs11671705	T	C
ALPI	rs117630211	G	A
ALPI	rs1409153	T	C
ALPI	rs9286381	T	C
ALPI	rs8176707	T	G
ALPI	rs567493	G	A
ALPI	rs2231862	T	C
ALPI	rs2431141	G	A
ALPI	rs35087747	T	G
ALPI	rs17369703	CAAAAAAAG	CAAAAAAAA
ALPI	rs10401347	A	G
ALPI	rs800245	A	G
ALPI	rs117410798	A	G
ALPI	rs62130341	T	C
ALPI	rs146798134	AATTGTGCCACTGCG	AATTGTGCCACTGCA
ALPI	rs112955485	T	G
ALPI	rs7035653	T	C
ALPI	rs62128046	T	C
ALPI	rs9411461	GG	CG
ALPI	rs11878193	G	C
ALPI	rs117608058	A	G
ALPI	rs35087293	TGTCTT	CGTCTT
ALPI	rs76426788	T	C
ALPI	rs140633800	GACGCCA	GACGCCG
ALPI	rs697356	C	G
CCDC134	rs5996067	A	G
CCDC134	rs28384790	C	G
CCDC134	rs11912852	T	G
CCDC134	rs8140869	A	G
CCDC134	rs139531	G	A
CCDC134	rs5758223	A	G

CCDC134	rs55906059	T	C
CCDC134	rs3804749	T	C
CCDC134	rs13053356	T	C
CCDC134	rs17002948	G	T
CCDC134	rs192077873	T	C
CCDC134	rs6002655	T	C
CCDC134	rs566607460	AACAACAACAAAAAA	AAAAACAACAAAAAA
CCDC134	rs76581697	T	C
CCDC134	rs147174547	A	G
CCDC134	rs149567399	T	G
CCDC134	rs73169175	G	T
CCDC134	rs112898596	T	C
CCDC134	rs28438468	G	A
CDH1	rs601338	A	G
CDH1	rs1236093	T	C
CDH1	rs635634	C	T
CDH1	rs560066139	C	G
CDH1	rs62132778	C	G
CDH1	rs12984035	T	C
CDH1	rs7044125	C	A
CDH1	rs4015	T	C
CDH1	rs75878619	A	C
CDH1	rs3094379	C	T
CDH1	rs374896	C	T
CDH1	rs201215552	T	G
CDH1	rs149181677	T	C
ST3GAL2	rs7137828	T	C
ST3GAL2	rs1265564	GAAAAAAAAAAC	GAAAAAAAAAAA
ST3GAL2	rs11065979	T	C
ST3GAL2	rs4766897	C	T
ST3GAL2	rs11066283	G	A
ST3GAL2	rs11066188	A	G
ST3GAL2	rs4378452	C	T
ST3GAL2	rs2057703	A	G

Table S5: Full results of the PheWAS-MR analysis for ALPI.

(This table is provided as a separate Excel file due to its length. See the file Table S5.xlsx.)
